# Leveraging User-Friendly Mobile Medical Devices to Facilitate Early Hospital Discharges in a Pediatric Setting: A Randomized Trial Study Protocol

**DOI:** 10.3390/children11060683

**Published:** 2024-06-04

**Authors:** Gianvincenzo Zuccotti, Marta Marsilio, Laura Fiori, Paola Erba, Francesca Destro, Costantino Zamana, Laura Folgori, Anna Mandelli, Davide Braghieri, Chiara Guglielmetti, Martina Pisarra, Letizia Magnani, Gabriele Infante, Dario Dilillo, Valentina Fabiano, Patrizia Carlucci, Elena Zoia, Gloria Pelizzo, Valeria Calcaterra

**Affiliations:** 1 Department of Biomedical and Clinical Science, University of Milan, 20157 Milan, Italy; d.braghieri1@campus.unimib.it (D.B.); valentina.fabiano@unimi.it (V.F.); gloria.pelizzo@unimi.it (G.P.); 2 Pediatric Department, Buzzi Children’s Hospital, 20154 Milan, Italy; laura.fiori@asst-fbf-sacco.it (L.F.); paola.erba@asst-fbf-sacco.it (P.E.); laura.folgori@asst-fbf-sacco.it (L.F.); dario.dilillo@asst-fbf-sacco.it (D.D.); patrizia.carlucci@asst-fbf-sacco.it (P.C.); valeria.calcaterra@unipv.it (V.C.); 3 Department of Economics, Management and Quantitative Methods, University of Milan, 20122 Milan, Italy; marta.marsilio@unimi.it (M.M.); chiara.guglielmetti@unimi.it (C.G.); martina.pisarra@unimi.it (M.P.); letizia.magnani@unimi.it (L.M.); gabriele.infante@unimi.it (G.I.); 4 Pediatric Surgery Department, Buzzi Children’s Hospital, 20154 Milan, Italy; francesca.destro@asst-fbf-sacco.it (F.D.); costantino.zamana@asst-fbf-sacco.it (C.Z.); 5 Intensive Care Unit, Buzzi Children’s Hospital, 20154 Milan, Italy; anna.mandelli@asst-fbf-sacco.it (A.M.); elena.zoia@asst-fbf-sacco.it (E.Z.); 6 Department of Internal Medicine, University of Pavia, 27100 Pavia, Italy

**Keywords:** user-friendly mobile, medical devices, early hospital discharges, pediatrics, children

## Abstract

Background: Mobile technology is increasingly prevalent in healthcare, serving various purposes, including remote health monitoring and patient self-management, which could prove beneficial to early hospital discharges. Aims: This study investigates the transitional care program experience facilitating early discharges in a pediatric setting through the use of an easy-to-use mobile medical device (TytoCare™, TytoCare Ltd., Natanya, Israel). Outcomes: This study aims to assess the effectiveness of telehomecare in achieving complete resolution of diseases without readmission, compare the length of stay between intervention and standard care groups, and gather user and professional experiences. Methods: A randomized open-label, controlled pilot study enrolled 102 children, randomly assigned to the telehomecare (TELE) group (*n* = 51, adopting early hospital discharge with continued home monitoring) or the standard-of-care (STAND) group (*n* = 51). Primary outcomes include complete disease resolution without readmission. Secondary objectives include recording a shorter length of stay in the intervention group. Surveys on user and professional experiences were conducted. A group of 51 children declining telemedicine services (NO-TELE) was also included. Results: In the TELE group, 100% of children achieved complete disease resolution without readmission, with a median duration of stay of 4 days, significantly shorter than the 7 days in the STAND group (*p* = 0.01). The telemedicine system demonstrated efficient performance and high satisfaction levels. The NO-TELE group showed no significant differences in demographics or digital technology competence. Perceived benefits of telemedicine included time and cost savings, reduced hospital stays, and technology utility and usability. Conclusions: This study demonstrates that user-friendly mobile medical devices effectively facilitate early hospital discharges in a pediatric setting. These devices serve as a bridge between home and hospital, optimizing care pathways.

## 1. Introduction

The utilization of digital health in pediatric care offers an opportunity to leverage technology for connecting patients and families with expert healthcare providers, mitigating costs and risks associated with hospital-based care [1]. Connected health, encompassing telecare, telemedicine, telehealth, m-Health, e-Health, and digital health services, has experienced substantial growth in pediatrics, addressing healthcare access disparities and overcoming social and geographic barriers [2,3].

As the shift toward home-based care supported by technology continues, patients and family caregivers need to understand and assume greater responsibilities for this new care model [4,5].

Recently, innovative “hospital at home” (HaH) initiatives have been developed to connect home-based care with hospital services, utilizing Technology-Enabled Care (TEC) for improved patient care and self-management. Kanagala et al. [6] reported that HaH care can result in lower median length of stay and readmission rates, offering a flexible model adaptable to varying demand situations with better clinical outcomes. Beyond economic considerations, early hospital discharge is crucial for optimizing care, especially in pediatric settings. The “European Association for Children in Hospital Charter” emphasizes the necessity of hospital admission only when essential, advocating for the prompt discharge of hospitalized children to ensure their psychophysical well-being [7].

Mobile technology is increasingly prevalent in healthcare [8,9], serving various purposes, including remote health monitoring and patient self-management, which could prove beneficial for early hospital discharges with significant psychosocial and economic impacts.

A user-friendly device refers to a device that is easy and intuitive to use, designed to accommodate the needs of a diverse range of users, including those with limited abilities or technological skills. A user-friendly mobile device, the TytoCare™ system, functions as both an otoscope and stethoscope [10,11,12]. Enabling remote examinations of ears, throat, skin, heart, and lungs, it supports the transmission of examination data from caregivers or healthcare professionals to physicians, overcoming obstacles to telemedicine integration.

Although limited, existing data on user-friendly mobile device use in pediatrics show promise. Wagner et al. [13] demonstrated concordance between measurements from remote physical examinations with a mobile medical device and in-person examinations in children older than 2 years. McDaniel et al. [14] highlighted superior sound and image quality compared to stand-alone devices, reducing diagnostic limitations. Notario et al. [15] demonstrated the feasibility and positive reception of in-home telehealth mobile devices, leading to reduced hospitalizations compared to usual care.

This study aims to explore the experience of a transitional care program using a user-friendly mobile medical device (TytoCare™, TytoCare Ltd., Natanya, Israel) to facilitate early hospital discharges in a pediatric environment. User and physician experience surveys will contribute valuable insights, furthering our understanding of the role of mobile medical devices as enablers in the perspective of HaH healthcare in pediatrics.

## 2. Methods

### 2.1. Study Design and Randomization

Before the start of a study, researchers should develop a detailed randomization protocol outlining the procedures for assigning participants to different treatment groups.

This study employed a single-center, two-group, randomized, open-label design with parallel arms conducted at Buzzi Children’s Hospital, situated in the metropolitan area of Milan, Italy. The experimental group involved early discharge with telehomecare (TELE), while the standard care (STAND) group received in-person physical care until the completion of hospitalization.

All patients/caregivers admitted from November 2022 to July 2023 who met the eligibility criteria were offered participation in the project. Those who agreed to engage in telemedicine were enrolled and subjected to randomization.

Informed consent was obtained and the allocation sequence, which was generated using a randomization procedure, assigned the participants to different groups. The randomization was executed in a 1:1 ratio (TELE/STAND) through a computer-generated number determinant. A physician who was unrelated to the process created a registration form to document it. Treatment allocation remained unblinded to both subjects and physicians.

**Figure 1:**
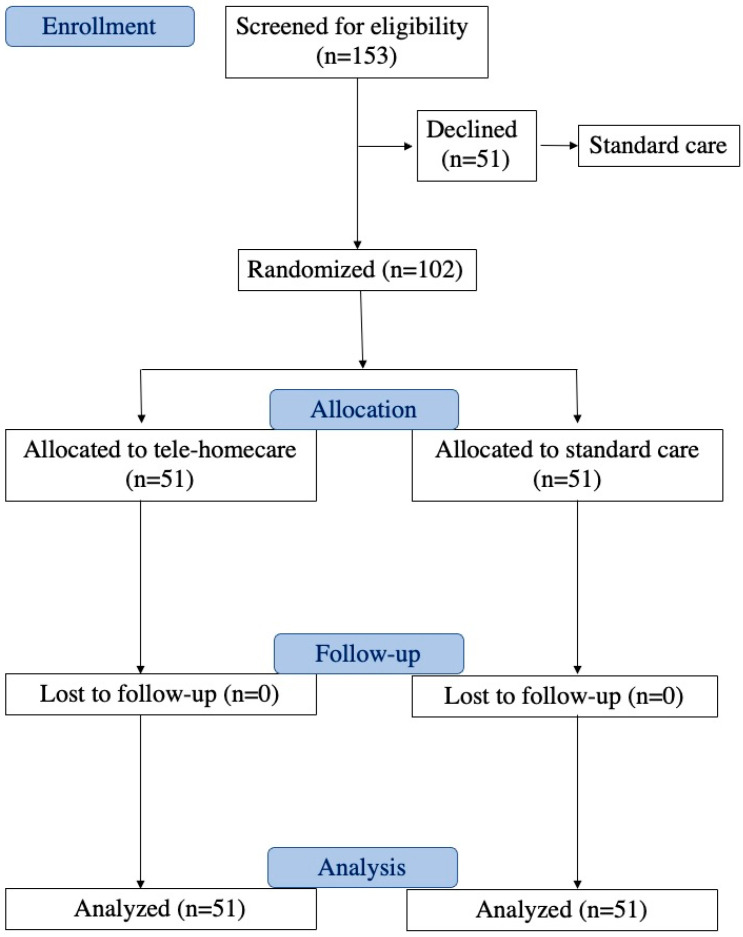
Flow diagram depicting the progression through the phases of the randomized trial involving two groups (telehomecare and standard care).

Children declining participation in the telemedicine project continued with standard treatment. The study process adhered to the Consolidated Standards of Reporting Trials guidelines, as illustrated in Figure 1. The protocol was registered in the Clinical Trials Registry clinicaltrials.gov (NCT06171763) (accessed on 20 December 2023).

Periodic review of documentation and verification of data integrity were scheduled during the study.

### 2.2. Participants

Participants in the present study were consecutively enrolled at the Buzzi Children’s Hospital (Milano, Italy) in the Pediatric Unit, Pediatric Surgery Unit, and Palliative Care Unit, and dyads of the hospitalized patient and family caregiver were recruited.

Inclusion criteria:

- Age of enrolled subjects: 0–18 years.
- Gender of patients (males and females).
- Patient status: hospitalized at the completion of treatment.
- Stability in vital signs (heart rate, respiratory rate, oxygen saturation).
- Stability/improvement/resolution in biochemical tests.
- No fever.
- Consent/Assent: participants must be willing and provide appropriate consent or assent based on their age.
- Proximity to domicile: living within a maximum 45 min distance from the facility.
- Adequate home environment
- Language proficiency: adequate proficiency in the Italian language.
- Possession of a compatible device.

Exclusion criteria:

- Refusal to participate in the program.
- Instability in vital signs.
- Presence of fever.
- Deteriorating results in biochemical tests.
- Living more than 45 min away from the facility.
- Inadequate home facilities.
- Language barrier.
- Lack of possession of a compatible device.
- Not having a device with an operating system capable of supporting the Tytocare app 7.0.0.433.

Parental consent was obtained from all participants or their responsible guardians after a thorough explanation of the study’s purpose. The study was conducted in accordance with the Declaration of Helsinki guidelines and approved by the Ethics Committee Milano Area 1 (Protocol number n. 0033846, date 3 August 2022). A medical team of 14 professionals were trained in the use of the Tytocare^TM^ device and voluntarily agreed to participate in the project.

### 2.3. Intervention

The telehomecare intervention entails an early hospital discharge with ongoing home monitoring utilizing a user-friendly mobile device (TytoCare™). Additionally, an in-person clinical reassessment was conducted 72 h after discharge to evaluate intervention outcomes.

Patient instructions were developed and shared to facilitate access to and download of medical reports, promoting patient engagement and empowerment in managing their healthcare.

#### 2.3.1. Experimental Group: Telehomecare

For patients/caregivers allocated to the TELE group, healthcare personnel trained in TytoCare™ system usage provided instructions before discharge. Subsequently, each patient received a device for use until the scheduled post-discharge clinical assessment. A parent or caregiver was invited to participate for each pediatric patient. 

Every 24 h, remote synchronous teleconsultation assessed the patient, with the physician using the TytoCare™ device for a comprehensive routine procedure, including medical history and physical examination. Data collection sheets were completed during the tele-visit. 

At the 72 h post-discharge mark, an in-person clinical assessment was scheduled to evaluate outcomes. Specifically, the visit evaluated the complete resolution of the disease state through a post-discharge objective examination.

TytoCare™ is a registered medical device compliant with European Medical Device Directive 93/42/EEC. The certificate of conformity and technical datasheet are provided in Supplementary Material S1. 

The TytoCare™ System served as the remote physical examination tool. This device enabled patients to conduct examinations and transmit audio, video, or image data to medical professionals located remotely; the TytoCare™ Device establishes a connection with an application on the patient’s mobile device to facilitate the communication of examination data and enable online meetings with clinicians. TytoCare™ is a modular, all-in-one device that encompasses functions such as a stethoscope, otoscope, tongue depressor, and thermometer:

- Stethoscope: Frequency range of 20–3500 Hz, heart rate range of 30–250 BPM, dimensions of 40 × 39 mm, and a weight of 0.06 kg.
- Otoscope: Image resolution of 640 × 480 (VGA), weight of 0.02 kg, and an adaptable speculum for children (3 mm).
- Tongue Depressor: For children (60 mm), weight of 0.011 kg.
- Thermometer: Detection range of 34.4–42.2 °C; accuracy of 0.2 °C for the temperature range 38–41 °C, with a precision of 0.2 degrees Celsius within the range of 38 to 41 degrees Celsius and a precision of 0.3 degrees Celsius outside this range (compliant with ASTM E1965-98 [16] and ISO 80601-2-56 [17]).

#### 2.3.2. Non-Intervention Group: Standard Care

Patients in the STAND group remained hospitalized for ongoing treatment. Every 24 h, in-person assessments by medical staff included a traditional physical examination utilizing standard equipment such as a digital thermometer, conventional stethoscope, and otoscope. The same data collection sheet was completed during these assessments. After 72 h of hospital observation, an in-person clinical examination was conducted to evaluate outcomes, specifically assessing the complete resolution of the disease state through a post-discharge objective examination.

Additionally, a group of 51 children declining telemedicine service (NO-TELE) was included in the study to record the reasons for refusal.

### 2.4. Outcomes

Considering in-person visits as the standard care procedure, the primary objective is to achieve concordance with complete resolution of the disease through in-person physical care without readmission to the hospital, expecting non-readmission in a minimum of 90% of cases. Secondary objectives include recording a lower length of stay in the interventional group. User and professional experience surveys were conducted to evaluate the level of acceptance and satisfaction with the telehomecare model.

### 2.5. Sample Size

A sample size of 50 in each randomized group achieves 80% power to detect a non-inferiority margin difference between group proportions of −0.0800. The reference group proportion is 0.9800, and the interventional group proportion is assumed to be 0.9000 under the null hypothesis of inferiority. The power was computed for the case when the standard treatment group proportion is 0.9889. The test statistic used is the one-sided Score test (Farrington and Manning). The significance level targeted for the test was 0.0500, and the achieved significance level by this design is 0.0465.

### 2.6. User and Physician Survey Questions

After randomization, in both the TELE and STAND groups, a user survey was distributed (see Table 1). The survey consisted of self-administered questions categorized into five sections, covering the following topics:

1. Sociodemographic information about the children and parents.
2. Employment details of parents who work.
3. Distance between home and the hospital.
4. Parents’ knowledge, attitudes, and technological skills in utilizing technologies, digital communication systems, and telehealth services [18,19,20].

Additionally, a survey on the perceived disadvantages and advantages of using the telemedicine option was recorded (see Table 2) [18,19,20].

Exclusively within the TELE group, supplementary data were gathered post-intervention to assess user satisfaction and the perceived level of care utilizing the Tytocare device (see Table 3).

In the TELE and STAND groups, the surveys were handed out immediately after randomization and then collected at the 72 h point during in-person clinical assessment.

Within the group of patients who declined the telemedicine service, a self-administered survey was recorded to identify factors that might have impeded their engagement and utilization of the proposed technological tools (see Table 4).

In all patients, a self-administered survey on the satisfaction level of the hospitalization experience was also recorded (see Table 5).

A physician experience survey was also self-administered at the conclusion of the study and was taken into consideration for this study’s purpose (see Table 6).

## 3. Data Analysis

Quantitative data were summarized using either mean and standard deviation (SD) or median and interquartile range (IQR), based on the data distribution assessed using the Shapiro–Wilk test. To test differences between groups, a Student *t* test or the one-way ANOVA were used. The significance was set at *p*-value less than 0.05. All data analyses were performed using Stata software version 16.1 (StataCorp, College Station, TX, USA).

## 4. Results

As reported in Figure 1, during the consecutive enrollment period, the project was presented to 153 hospitalized patients (86 males, 67 females, mean age 3.37 ± 4.0 years).

Among them, 102 patients accepted the telemedicine solution and were subsequently randomized, while the remaining 51 patients declined the telemedicine option.

### 4.1. Randomized Telehomecare and Standard Care Groups

A total of 102 children (59 males and 43 females, mean age of 3.36 ± 3.98 years), affected by respiratory diseases (56.9%) (bronchiolitis, pneumonia, asthma, upper airways disorders), abdominal pathologies (17.6%) (gastroenteritis, colitis, appendicitis, stones) and other pathologies (21.5%) (osteomyelitis, uro-genital and other infections, endocrine problems, burns) accepted the telemedicine service.

These patients were randomly assigned to the TELE group (*n* = 51) or the STAND group (*n* = 51), as illustrated in Figure 1.

#### 4.1.1. Clinical Outcomes

In a comparable manner to the STAND group, a total of 51/51 (100%) children included in the TELE arm reached complete resolution of disease during the telemedicine intervention, without readmission to the hospital.

An adequate transmission of clinical parameters with mobile devices was obtained in all children. No significant technical problems occurred during telemonitoring (Table 3).

With home telemonitoring, the median length of hospital stay was 4 days (IQR 3–8) in the interventional group, significantly shorter than 7 days (IQR 5–9) in the STAND group (*p* = 0.01).

#### 4.1.2. User Survey Results in Randomized Groups

All enrolled participants successfully completed the survey. Table 1 illustrates the survey questionnaire along with the corresponding responses.

The TELE and STAND groups exhibited no significant difference in terms of sociodemographic characteristics, as well as the parents’ proficiency in utilizing technologies, digital communication systems, and telehealth services.

In both groups, in most cases, the survey was completed by the mother (TELE: 84.32%, STAND: 94.12%) who had a regular job (TELE: 72.5%, STAND: 76.47%).

Respondents generally had a high level of education (TELE: 86.0%, STAND: 92.16%) and demonstrated excellent or good competence in using digital technologies (82.35%). Internet access at home was usually used every day (100%). Only 13.73% of cases in TELE and 8.33% in STAND had previous telemedicine experience.

**Figure 2:**
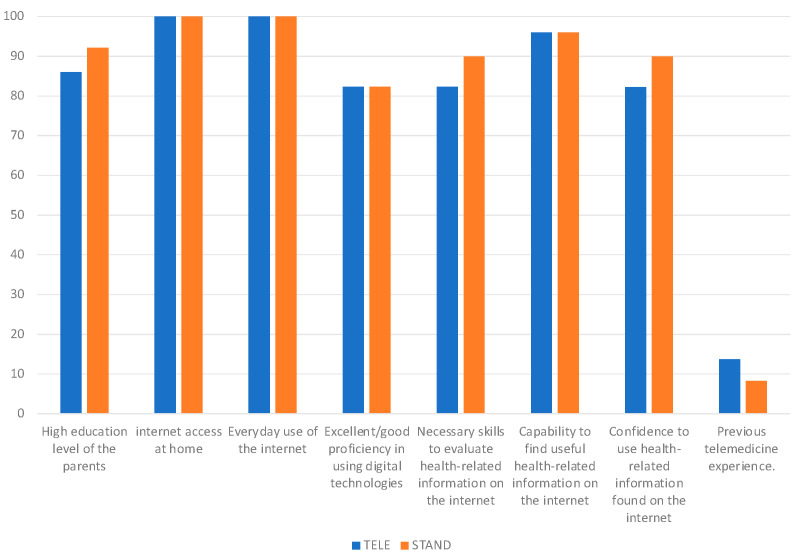
Parents’ knowledge, attitudes, and technological skills in utilizing technologies, digital communication systems, and telehealth services.

In Figure 2, the main knowledge, attitudes and skills of the parents in using technologies, digital communication systems and telehealth services are shown.

In Table 2, the disadvantages and advantages of using telemedicine options are reported. In both groups, the main possible limitation of telemedicine is the lack of personal contact with doctors, and in more than 80% of cases, a tele-visit was not considered as reliable as a real visit.

**Figure 3:**
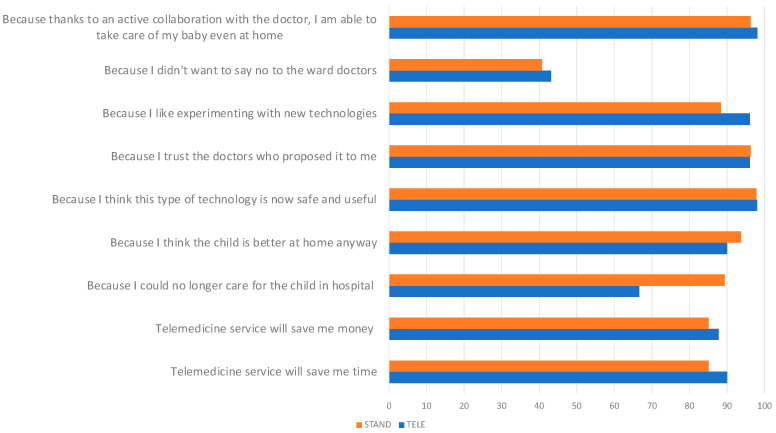
Motivations for using telemedicine.

As reported in Figure 3, the main motivations for using telemedicine included time (90%) and money in travel and permits (87.76%), relevance to stay at home (90%), technology safety and utility (98%), significant trust in the professionalism of the service providers (96.9%).

In Table 3, the satisfaction survey for the TELE group in using the telemedicine option is presented. The TELE users were very satisfied or satisfied in all areas on training in using, including operational steps (91.49%), contact (91.49%), therapeutic indications (87.24%), support (89.13%).

All respondents reported satisfaction with the telemedicine experience (100%); no dissatisfaction was expressed (0%). In the 97.87% of cases, parents are able to correctly describe the child’s health information, sharing the parameters without technical problems (100%). Additionally, 93.75% of the respondents stated they would repeat the telemedicine experience.

### 4.2. No-Telemedicine Group

A total of fifty-one patients (27 males and 24 females), affected by respiratory diseases 64.7%, abdominal pathologies 13.7% and other disorders 21.6%, with a mean age of 3.47 ± 4.04 years, comprised the NO-TELE group.

There is no statistically significant difference in demographic data and types of diseases between the group that declined the telemedicine option and the groups that accepted the telemedicine solution. All patients achieved full recovery from the disease during their hospitalization.

Table 1 and Table 3 present the survey questionnaire along with the corresponding responses of patients in the NO-TELE group.

Upon comparison with the group that embraced the telemedicine option, no significant differences were observed in terms of age and demographic features. The majority demonstrated excellent or good competence in using digital technologies (78%) and reported regular daily Internet use at home (97.56%).

The primary reasons for declining the telemedicine option included the inability to establish a personal relationship with the medical doctor (95.45%) and the incapacity to ask the medical doctor questions directly (93.18%). Respondents expressed concerns about the reliability of tele-consultations compared to in-person visits (97.73%) and showed a preference for traditional hospitals as the optimal care structure (97.83%). Worries about privacy issues (68.89%), difficulties in using technology (84.09%), and potential technical problems (68.18%) were also cited as limiting factors. Despite the current refusal of the telemedicine option, 40.82% of cases did not rule out the possibility of using the service in the future.

### 4.3. Patient Satisfaction Level of the Hospitalization Experience

In Table 4, the survey results on the hospitalization experience in all groups are presented. In all, the level of patient satisfaction is high, with no significant differences observed between the groups.

### 4.4. Physician Survey Results

As reported in Table 6, 96.15% of physicians expressed satisfaction with the adopted system. During tele-visits, no or limited technical problems, immediately resolved (temporary audio problems), were detected in 78.85% of cases; in the remaining 21.15%, temporary connection problems were recorded. Comfortable communication (100%) and useful data sharing with parents (94.23%) were reported, with no issues in following-up instructions (94.24%) and with adequate adherence to the agreed-upon time (98.08%).

## 5. Discussion

Our study underscores the utility of user-friendly mobile medical devices, exemplified by the TytoCare™ system, in effectively reducing hospital stays while achieving optimal clinical outcomes comparable to standard care. The system showed proficient performance in transmitting clinical information during telehealth visits, earning high levels of satisfaction from both patients and physicians. These mobile medical devices serve as a valuable bridge between home and hospital, optimizing care pathways.

Hospitalization can be an inherently stressful experience for children, impacting behavioral and emotional responses with potential implications for health and developmental outcomes [21]. Hospitalized children may undergo a disconnection from their familiar family surroundings and recreational activities, navigating an environment where they encounter unfamiliar and invasive medical procedures [22]. These alterations can be perceived as traumatic events, eliciting stress responses. In certain instances, susceptibility to stress, clinical conditions, and care-related characteristics may contribute to the onset of Pediatric Medical Traumatic Stress, resulting in enduring negative psychological effects [23,24]. The risks associated with these stressors are particularly pronounced in cases of chronic illnesses, conditions involving frequent or prolonged hospitalizations, or in the presence of psychosocial vulnerability [25]. Furthermore, the hospitalization of a child is a stressful event for parents as well [24]. Parents often grapple with anxiety and depression during the hospitalization period, especially in families with children facing chronic pathologies [26,27], leading to significant implications for the child’s health and behavioral outcomes [24,26,27].

Improving the hospital discharge process as highlighted by the “European Association for Children in Hospital Charter” [7] is crucial in pediatric care, aligning with the imperative to discharge children promptly for their psychophysical well-being [28]. Telemedicine, as a bridge between home and hospital, emerges as a promising tool, though the literature on its role in early hospital discharge is limited [29,30]. Vesterby et al. [29] reported that telemedicine support shortens length of stay after fast-track hip replacement in adults. Minguez Clemente et al. [31] demonstrated that follow-up through a telemedicine program after early discharge from hospitalization is equally effective as conventional home follow-up in patients with chronic obstructive pulmonary disease. Our study contributes valuable insights by demonstrating a significant reduction in hospital stays through the use of a mobile medical device in a pediatric setting. This suggests that a telemedicine approach allows for quicker discharges without compromising clinical safety or physical outcomes, thereby enhancing the quality of children’s care.

Ensuring timely discharge and preventing readmissions are crucial indicators of effective integration between hospital and community services [32]. The use of a user-friendly mobile medical device not only led to a reduction in hospital stays but also ensured satisfactory clinical outcomes without instances of readmission, supporting the effectiveness of home telecare. This highlights the role of mobile remote presence devices as integrative tools for remote patient monitoring, supporting the evolution of family-centered care [30]. A hybrid “home–hospital” model could be seen as a prospective approach in pediatric care for “vulnerable” children, including those with chronic illnesses and medical fragility.

These populations, which have a high priority for health services and technological support systems, could greatly benefit from this approach to address their multiple health needs [8].

Our study participants expressed satisfaction with TytoCare^TM^, even if they were initially unfamiliar with the concept. The positive experiences reported, coupled with a willingness to recommend the service, emphasize the potential of these technologies in healthcare. Trust in professional service emerged as a crucial factor influencing user satisfaction and perceived care levels, underscoring the need for integrating technological innovation into medical training, as emphasized by Chastonay et al. [33].

Perceived benefits of telemedicine, including time and cost savings, reduced hospital stays, and technology utility and usability, align with the evolving landscape of healthcare delivery. While limitations were reported by some individuals opting against telemedicine, our findings overall support the positive impact of mobile medical devices in pediatric care, fostering patient satisfaction and efficient healthcare delivery. The telemedicine option leads to a lack of personal contact with one’s medical doctor during telemedicine services. These results confirm that some disadvantages of telehealth include limitations with performing comprehensive physical examinations with impersonal interactions with physicians [3].

Telemedicine must deal with many legal and regulatory obstacles [9]. Even though our users expressed limited privacy concerns, regulatory frameworks for telemedicine are insufficient, often lacking clarity [3]. The ethical responsibilities, conflict of interest considerations, and obligations to protect personal health information are the same for practicing telemedicine as they are for practicing in-person medicine; thus, regulatory, legal, and ethical considerations of telemedicine should be taken into account. However, at this time, particularly concerning healthcare and medical practice, uniform regulations at the European level are absent [3]. The wide range of norms and regulations governing practice and privacy contributes to confusion for providers involved in telemedicine practice [3].

According to the literature [34], we recorded high levels of physician satisfaction utilizing telemedicine. Telemedicine utilizing mobile devices can serve as an additional tool to facilitate a broader and more enduring adoption of telemedicine [34].

To fully harness the potential of telemedicine and remove the obstacles to its utilization, future research and initiatives are imperative. These should focus on enhancing the long-term satisfaction in using telemedicine and the cost-effectiveness of telemedicine services, ensuring unrestricted access to the internet, and establishing specific guidelines concerning data protection safety, informed consent, and professional liability [8].

## 6. Study Limitations

As a pilot study, this research was limited by the small sample size with various diseases at admission; thus, further studies with a larger number of patients are mandatory to confirm the positive role of user-friendly mobile medical devices in early hospital discharges, investigating potential variations among age groups (neonates, infants, children, and adolescents) and types of disease.

We present a randomized study to explore a transitional care program utilizing a user-friendly mobile medical device. Randomization alone did not completely remove the potential for systematic differences between treatment groups in this study. Enthusiasm for a new treatment may lead to improved outcomes being observed in this patient group, irrespective of the actual treatment efficacy. Thus, a blinded or masked trial can help limit bias. However, in some cases, such as in our protocol, the nature of the treatments under investigation makes blinding difficult. Therefore, to mitigate bias related to the open-label design, we applied a meticulous study design and randomization to patients/caregivers who agreed to participate in telemedicine. All steps of the protocol and randomization have been planned and recorded to ensure transparency at every stage, thereby facilitating reproducibility. Additionally, standardized eligibility criteria were applied before randomization.

Additionally, in order to better evaluate user acceptance, additional aspects of user and physician satisfaction should be explored further, also considering that the final timeline of the project and the availability of users and professionals to participate can certainly impact the reported satisfaction levels. The long-term use of the system will help us better understand the factors that most significantly influence the level of satisfaction and adaptability of this technological approach into existing processes, workflows, and practices.

Finally, the viability of the telemedicine service needs to be assessed in relation to cost–benefit considerations, considering both direct and indirect costs for patients, families, organizations, and the healthcare system [3,27,34,35]. While this project is currently in the exploratory phase, economic evaluation has not yet been incorporated but is planned as a future objective. Specifically, we will evaluate the sustainability of the telemedicine approach, taking into consideration the value that it can generate in social, economic–administrative, and environmental contexts, while considering both the professional/hospital context and that of the user.

## 7. Conclusions

In conclusion, our study supports the idea that user-friendly mobile medical devices may provide a valuable tool for facilitating early hospital discharges in a pediatric setting. This approach not only maintains a high level of care but also effectively reduces the length of stay, enhancing the quality of children’s care. The integration of mobile medical devices represents a significant advancement in clinical tools to facilitate family-centered care for children, aligning with the vision of HaH healthcare and the concept of a virtual public hospital in pediatrics.

## Figures and Tables

**Table 1:** Telehomecare and standard care user survey.

Question	Telehomecare	Standard Care	No-Telemedicine
Demographic and socioeconomic characteristics of the children and parents			
Who is completing the questionnaire?			
Mother	84.31%	94.12%	86.27%
Father	15.69%	5.88%	11.76%
Other	0%	0%	1.96%
What is the age of the individual completing the questionnaire?	35.70 ± 10.79	35.93 ± 7.81	38.08 ± 8.47
What is the nationality of the individual completing the questionnaire?			
		
Italian/Foreign	72.6%/27.4%	70.59%/29.41%	82.35/17.65%
Does the patient have siblings?			
Yes (1/more than 1)	52%	53.33%	54.9%
	(38%/14%)	(30.0%/16.67%)	(37.25%/17.4%)
No	48%	53.33%	45.1%
What is the parental educational background of the individual answering the questionnaire?			
Middle school	12.0%	10.0%	7.84%
High school	46.0%	40.0%	52.94%
College and/or postgraduate	42.0%	50%	39.21%
Employment characteristics of the working parents			
Is the mother currently employed?			
Yes, employed-full-time/part-time/independent contractor	72.5%	76.47%	78.43%
No, not seeking work/unemployed and seeking work	27.45%	23.53%	21.57%
Is the father currently employed?			
Yes, employed-full-time/part-time/independent contractor	97.877%	100%	96.08%
No, not seeking work/unemployed and seeking work	2.13%	0%	3.92
Distance from residence to hospital			
How far is the hospital from your home (in kilometers)?	26.25 ± 10.40	24.65 ± 16.65	25.81 ± 10.79
What mode of transportation would you use to reach the hospital?			
Public transport/Private vehicle	16.33%/83.67%	19.57%/80.43%	8.7%/92.30%
Parental proficiency, mindset, and ability in utilizing technology, digital communication systems and telehealth services			
How frequently do you:			
Require assitance with reading medical documentation			
- never/seldom	25.49%/33.33%	16.67%/23.33%	11.76%/21.57%
- sometimes	29.41%	46.67%	45.10%
- often/always	9.8%/1.96%	13.33%/0%	19.61%/1.96
Experience challenges in comprehending health status because of reading limitations			
- never/seldom	27.45%/37.25%	16.67%/30%	17.65%/21.57%
- sometimes	27.45%	43.33%	50.98%
- frequently/always	4%/0%	6.67%/3.33%	9.80%
Encounter difficulties in comprehending health-related information			
- never/seldom	39.22%/37.25%	23.33%/33.33%	25.49%/37.25%
- sometimes	19.61%	40.0%	29.41%
- frequently/always	3.92%/0%	0%/3.33%	7.84%
Feel confident in filling out medical consent forms			
- never/seldom	20.0%/16.0%	10.0%/6.67%	11.76%/21.57%
- sometimes	20%	30%	45.10%
- frequently/always	36%/8%	30%/23.33%	19.6%/1.96%
Do you have internet access at your home?			
Yes/No	94.12%/5.88%	93.33%/6.67%	89.90%/10.20%
How frequently do you utilize your home internet connection?			
Every day	100%	100%	97.56%
One/two times a week	0%	0%	2.44%
Occasionally	0%	0%	0%
What do you primarily use your smartphone for?			
Phone calls	94.11%	94.12%	94.11%
Messages	92.16%	902%	88.23%
Shopping	72.55%	82.36	68.62%
Banking	76.47%	74.5%	64.71%
Sending emails	90.20%	86.28	74.51%
Learning	52.94%	54.90%	47.06%
Social network use	72.55%	58.8%	66.66%
Entertainment (games/movies)	62.74%	62.74%	49.01%
Checking health status	39.21%	43.14	68.63%
How would you rate your proficiency in using digital technologies?			
Excellent/Good	82.35%	82.35%	78%
Moderate	17.65%	17.65%	20%
Inadequate	0%	0%	2%
Utilization of applications or online platforms for:			
- scheduling appointments	80.97%	80.39%	84.31%
- accessing medical records	80.39%	80.39%	78.43%
- transmitting medical documents or images through email	82.35%	82.35%	70.58%
- interacting with healthcare staff or posing inquiries	80.39%	80.39%	68.63%
- electronic prescriptions	74.51%	74.50%	82.35%
- exploring health-related data	74.51%	74.50%	74.51%
- other purposes	0.04%	0.04%	0.02%
I possess the ability to locate valuable health-related content online			
Fully agree/Agree/Tend to agree	96.08%	96%	98%
Disapprove	3.92%	4%	2%
I am proficient in assessing health-related information obtained from the internet			
Fully agree/Agree/Tend to agree	82.35%	90%	86%
Disapprove	17.65%	10%	14%
I am comfortable utilizing health-related information sourced from the internet.			
Fully agree/Agree/Tend to agree	86.27%	90%	80%
Disapprove	13.73%	10%	20%
I began utilizing the internet to seek health-related information only following the onset of the COVID-19 pandemic			
Fully agree/Agree/Tend to agree	52%	41%	46%
Disapprove	48%	59%	54%
Tele-visit			
Did you know the definition of “Tele-visit” prior to today?			
Yes	45.10%	20%	50.98%
No	54.90%	78%	47.06%
I thought it was something different	0%	2%	1.96%
Have you ever used a telemedicine service in the past?			
Yes/No	13.73%/86.27%	8.33%/91.67%	6%/94%
If Yes, regarding your previous experiences with telemedicine, what is your level of satisfaction?			
Very satisfied/Satisfied/Partially satisfied	100%	96%	95.45%
Not at all satisfied	0%	4%	4.55%

**Table 2:** Disadvantages and advantages of using telemedicine option.

Question	Telehomecare	Standard Care
Disadvantages and limits		
Because I believe that you don’t establish a personal relationship with the doctor		
Fully agree/Agree/Tend to agree	82.0%	85.59%
Disapprove	18.0%	14.41%
Because I believe that it is not possible to ask the doctor all the questions		
Fully agree/Agree/Tend to agree	75.51%	82.14%
Disapprove	24.49%	17.86%
The proposed technological tools are too difficult to use		
Fully agree/Agree/Tend to agree	78.43%	77.78%
Disapprove	21.57%	22.22%
I think a tele-visit is NOT as reliable as a real visit		
Fully agree/Agree/Tend to agree	86.27%	92.86%
Disapprove	13.73%	7.14%
I think I might have connection problems		
Fully agree/Agree/Tend to agree	66.7%	50%
Disapprove	33.3%	50%
I’m not familiar enough with technology in general		
Fully agree/Agree/Tend to agree	68.63%	60.71%
Disapprove	31.37%	39.29%
I would still need someone’s help during the visit (for connection, to hold the baby, etc.) and it’s not guaranteed that they will be available		
Fully agree/Agree/Tend to agree	68.63%	67.86%
Disapprove	31.37%	32.14%
I fear there may be privacy issues		
Fully agree/Agree/Tend to agree	54.9%	64.29%
Disapprove	45.10%	35.71%
Main reason why you decided to make yourself available to adopt the telemedicine service for your child’s discharge		
It will save me time		
Fully agree/Agree/Tend to agree	90.0%	85.11%
Disapprove	10.0%	14.89%
It will save me money (travel, permits, etc.)		
Fully agree/Agree/Tend to agree	87.76%	85.11%
Disapprove	12.24%	14.89%
Because I could no longer care for the child in hospital (due to work, caring for other children, etc.)		
Fully agree/Agree/Tend to agree	66.7%	89.36%
Disapprove	33.33%	10.64%
Because I think the child is better at home anyway		
Fully agree/Agree/Tend to agree	90.0%	93.75%
Disapprove	10%	6.25%
Because I think this type of technology is now safe and useful		
Fully agree/Agree/Tend to agree	98.0%	97.87%
Disapprove	2.0%	2.13%
Because I trust the doctors who proposed it to me		
Fully agree/Agree/Tend to agree	96.08%	96.3%
Disapprove	3.92%	3.7%
Because I like experimenting with new technologies		
Fully agree/Agree/Tend to agree	96.08%	88.4%
Disapprove	3.92%	11.54%
Because I didn’t want to say no to the ward doctors		
Fully agree/Agree/Tend to agree	43.14%	40.74%
Disapprove	56.86%	59.26%
Because thanks to an active collaboration with the doctor, I am able to take care of my baby even at home		
Fully agree/Agree/Tend to agree	98.04%	96.3%
Disapprove	1.96%	3.70%

**Table 3:** Satisfaction survey in using telemedicine in telehomecare group.

Questions	Telehomecare
The information received upon discharge was clear regarding:	
Operation of Tytocare device	
No, not at all	2.13%
Yes, but not as clear	6.38%
Yes/Yes how much	91.49%
Who would have contacted her	
No, not at all	2.13%
Yes, but not as clear	6.38%
Yes/Yes how much	91.49%
When he would contact her	
No, not at all	2.13%
Yes, but not as clear	0%
Yes/Yes how much	97.87%
How Tytocare should have been used	
No, not at all	4.26%
Yes, but not as clear	8.51%
Yes/Yes how much	91.49%
How he should have followed the therapy	
No, not at all	8.51%
Yes, but not as clear	4.26%
Yes/Yes how much	87.24%
What to do if the child gets worse	
No, not at all	8.89%
Yes, but not as clear	4.44%
Yes/Yes how much	86.66%
Who to contact in case of need	
No, not at all	2.17%
Yes, but not as clear	8.70%
Yes/Yes how much	89.13%
Satisfaction in using telemedicine	
How satisfied are you overall with the telehomecare you received?	
No, not at all	0%
Yes/Yes how much	100%
During telehomecare the doctors who treated the child were very scrupulous and caring	
Fully agree/Agree/Tend to agree	96%
Disapprove	4%
The doctor made me feel safe while continuing the therapy at home with tele-visits	
Fully agree/Agree/Tend to agree	100%
Disapprove	0%
The doctor made the experience during the tele-visits pleasant	
Fully agree/Agree/Tend to agree	97.92%
Disapprove	2.08%
I felt comfortable communicating with the professional using the telemedicine system	
Fully agree/Agree/Tend to agree	97.87%
Disapprove	2.13%
I was able to describe my child’s health condition during televisit	
Fully agree/Agree/Tend to agree	97.87%
Disapprove	2.13%
I managed to collect and share the required parameters with the doctor	
Fully agree/Agree/Tend to agree	100%
Disapprove	0%
I followed all the instructions I was given on what to do once I got home	
Fully agree/Agree/Tend to agree	100%
Disapprove	0%
I had no technical problems during the tele-visit (e.g., connection, hearing, seeing)	
Fully agree/Agree/Tend to agree	100%
Disapprove	0%
How do you rate the telemedicine experience?	
Poor/Fair	0%
Good/very good	100%
In light of your experience with Tytocare, if you were asked to use the Tytocare telemedicine device in the future, do you think you would still be willing to evaluate its use?	
No/More no that yes	6.25%
Yes/More yes that no	93.75%

**Table 4:** Survey on reasons for rejecting the telemedicine option among non-telemedicine users.

Question	No Telemedicine
Main reason why you decided NOT to make yourself available to adopt the Tytocare device for your child’s discharge	
Because I believe that you don’t establish a personal relationship with the doctor	
Fully agree/Agree/Tend to agree	95.45%
Disapprove	4.55%
Because I believe that it is not possible to ask the medical doctor all the questions	
Fully agree/Agree/Tend to agree	93.18%
Disapprove	6.82%
The proposed technological tools are too difficult to use	
Fully agree/Agree/Tend to agree	84.09%
Disapprove	15.91%
I think a tele-visit is NOT as reliable as a real visit	
Fully agree/Agree/Tend to agree	97.73%
Disapprove	2.27%
I think I might have connection problems	
Fully agree/Agree/Tend to agree	68.18%
Disapprove	31.82%
I’m not familiar enough with technology in general	
Fully agree/Agree/Tend to agree	62.22%
Disapprove	37.78%
I would still need someone’s help during the visit (for connection, to hold the baby, etc.) and it’s not guaranteed that they will be available	
Fully agree/Agree/Tend to agree	69.57%
Disapprove	30.43%
I fear there may be privacy issues	
Fully agree/Agree/Tend to agree	68.89%
Disapprove	31.11%
Because I don’t trust technologies	
Fully agree/Agree/Tend to agree	65.22%
Disapprove	34.78%
I think that only in hospital does my son receive the best care	
Fully agree/Agree/Tend to agree	97.83%
Disapprove	2.17%
Parent’s willingness to use the device in the future	
No/More no that yes	59.18%
Yes/More yes that no	40.82%

**Table 5:** Patient satisfaction level of the hospitalization experience [9] in telehomecare, standard care and no-telemedicine groups.

Question	Telehomecare	Standard Care	No Telemedicine
The doctors who treated the child were very thorough and caring			
Fully agree/Agree/Tend to agree	96%	100%	100%
Disapprove	4%	0%	0%
The doctors attending to the child were exceptionally meticulous and compassionate			
Fully agree/Agree/Tend to agree	100%	100%	100%
Disapprove	0%	0%	0%
The parent believes that the child can only receive the best care in a hospital			
Fully agree/Agree/Tend to agree	Not considered	53.33%	50.98%
Disapprove		46.67%	49.02%
At times, the doctor treating the child did not pay attention to what the child or the caregiver was attempting to communicate			
Fully agree/Agree/Tend to agree	32%	40%%	50.98%
Disapprove	68%	60%%	49.02%
The parent believes that the doctor was not as competent as they should have been.			
Fully agree/Agree/Tend to agree	90%	100%	90%
Disapprove	10%	0%	10%
The parent is satisfied with the care received			
No	0%	0%	0%
Yes	100%	100%	100%
Would you recommend this department to other patients?			
No/More no that yes	0%	0%	0%
Yes/More yes that no	100%	100%	100%
Would you still choose this department for treatment?			
No/More no that yes	0%	0%	0%
Yes/More yes that no	100%	100%	100%
How would you rate the care you received?			
Poor/Fair	0%	0%	0%
Good/very good	100%	100%	100%

**Table 6:** Physician survey.

Questions	Response %
How satisfied are you overall with the Tytocare process with patients?	
- Not at all	0%
- A little	3.85%
- Somewhat	21.15%
- A lot	75%
How many adults were present during the televisit?	
- 1	65.22%
- >1	34.78%
How well were the parents able to follow all the instructions provided for home care?	
- Not at all	0%
- A little	5.77%
- Somewhat	9.62%
- A lot	84.62%
Did the parents adhere to the agreed-upon time for the televisit?”	
- Not at all	0%
- A little	1.92%
- Somewhat	11.54%
- A lot	86.54%
Were there any technical problems during the tele-visit?	
- Not at all	42.31%
- A little, immediately resolved	15.38%
- Somewhat, immediately resolved	21.15%
- A lot	21.15%
How successful was the parent in collecting and sharing the required information and parameters?	
- Not at all	1.92%
- A little	3.85%
- Somewhat	23.08%
- A lot	71.15%
How comfortable did you feel communicating with your parent using the telemedicine system?	
- Not at all	0%
- A little/Somewhat	23.08%
- A lot	76.92%

## Data Availability

The authors confirm that the data supporting the findings of this study are available within the article.

## Supplementary Materials

The following supporting information can be downloaded at https://www.mdpi.com/article/10.3390/children11060683/s1: Supplementary Material S1: Certificate of conformity and technical datasheet of TytoCare™.

## References

[1] Bird M., Li L., Ouellette C., Hopkins K., McGillion M.H., Carter N. (2019). Use of Synchronous Digital Health Technologies for the Care of Children with Special Health Care Needs and Their Families: Scoping Review. JMIR Pediatr. Parent..

[2] Taylor K. (2015). Connected health. How Digital Technology Is Transforming Health and Social Care.

[3] Gajarawala S.N., Pelkowski J.N. (2021). Telehealth Benefits and Barriers. J. Nurse Pract..

[4] Barbosa W., Zhou K., Waddell E., Myers T., Dorsey E.R. (2021). Improving Access to Care: Telemedicine Across Medical Domains. Annu. Rev. Public Health.

[5] Haleem A., Javaid M., Singh R.P., Suman R. (2022). Medical 4.0 technologies for healthcare: Features, capabilities, and applications. Internet Things Cyber-Physical Syst..

[6] Kanagala S.G., Gupta V., Kumawat S., Anamika F., McGillen B., Jain R. (2023). Hospital at home: Emergence of a high-value model of care delivery. Egypt. J. Intern. Med..

[7] McGillion M., Yost J., Turner A., Bender D., Scott T., Carroll S., Ritvo P., Peter E., Lamy A., Furze G. (2016). Technology-Enabled Remote Monitoring and Self-Management—Vision for Patient Empowerment Following Cardiac and Vascular Surgery: User Testing and Randomized Controlled Trial Protocol. JMIR Res. Protoc..

[8] Zuccotti G., Calcaterra V., Foppiani A. (2023). Present and future of telemedicine for pediatric care: An Italian regional experience. Ital. J. Pediatr..

[9] Mannarino S., Calcaterra V., Fini G., Foppiani A., Sanzo A., Pisarra M., Infante G., Marsilio M., Raso I., Santacesaria S. (2024). A pediatric telecardiology system that facilitates integration between hospital-based services and community-based primary care. Int. J. Med Inform..

[10] Fabbrizio A., Fucarino A., Cantoia M., De Giorgio A., Garrido N.D., Iuliano E., Reis V.M., Sausa M., Vilaça-Alves J., Zimatore G. (2023). Smart Devices for Health and Wellness Applied to Tele-Exercise: An Overview of New Trends and Technologies Such as IoT and AI. Healthcare.

[11] Haskel O., Itelman E., Zilber E., Barkai G., Segal G. (2022). Remote Auscultation of Heart and Lungs as an Acceptable Alternative to Legacy Measures in Quarantined COVID-19 Patients—Prospective Evaluation of 250 Examinations. Sensors.

[12] Wagner R., Lima T.C., da Silva M.R.T., Rabha A.C.P., Ricieri M.C., Fachi M.M., Afonso R.C., Motta F.A. (2023). Assessment of Pediatric Telemedicine Using Remote Physical Examinations with a Mobile Medical Device: A Nonrandomized Controlled Trial. JAMA Netw. Open.

[13] McDaniel N.L., Novicoff W., Gunnell B., Gordon D.C. (2019). Comparison of a Novel Handheld Telehealth Device with Stand-Alone Examination Tools in a Clinic Setting. Telemed. J. e-Health Off. J. Am. Telemed. Assoc..

[14] Notario P.M., Gentile E., Amidon M., Angst D., Lefaiver C., Webster K. (2019). Home-Based Telemedicine for Children with Medical Complexity. Telemed. J. e-Health Off. J. Am. Telemed. Assoc..

[15] http://www.aal-europe.eu/wp-content/uploads/2020/02/vINCI-Call-2017-DIGITAL-SKILLS-QUESTIONNAIRE-END-USERS.pdf.

[16] (2023). Standard Specification for Infrared Thermometers for Intermittent Determination of Patient Temperature.

[17] (2017). Medical Electrical Equipment—Part 2-56: Particular Requirements for Basic Safety and Essential Performance of Clinical Thermometers for Body Temperature Measurement.

[18] Vuorikari R., Kluzer S., Punie Y. (2022). DigComp 2.2: The Digital Competence Framework for Citizens—With New Examples of Knowledge, Skills, and Attitudes, EUR 31006 EN.

[19] Bravo G., Del Giudice P., Poletto M., Battistella C., Conte A., De Odorico A., Lesa L., Menegazzi G., Brusaferro S. (2018). Validazione Della Versione Italiana del Questionario di Alfabetizzazione Sanitaria Digitale (IT-eHEALS). Bollettino Epidemiologico Nazionale. https://www.epicentro.iss.it/ben/2018/luglio-agosto/2.

[20] Bonn M. (1994). The effects of hospitalisation on children: A review. Curationis.

[21] Gomes G.L.L., Fernandes M.d.G.M., da Nóbrega M.M.L. (2016). Hospitalization anxiety in children: Conceptual analysis. Ansiedade da hospitalização em crianças: Análise conceitual. Rev. Bras. Enferm..

[22] Al Jowf G.I., Ahmed Z.T., An N., Reijnders R.A., Ambrosino E., Rutten B.P.F., de Nijs L., Eijssen L.M.T. (2022). A Public Health Perspective of Post-Traumatic Stress Disorder. Int. J. Environ. Res. Public Health.

[23] Commodari E. (2010). Children staying in hospital: A research on psychological stress of caregivers. Ital. J. Pediatr..

[24] Oh D.L., Jerman P., Marques S.S., Koita K., Boparai S.K.P., Harris N.B., Bucci M. (2018). Systematic review of pediatric health outcomes associated with childhood adversity. BMC Pediatr..

[25] Coyne I. (2006). Children’s Experiences of Hospitalization. J. Child Health Care.

[26] Brazil K., Krueger P. (2002). Patterns of family adaptation to childhood asthma. J. Pediatr. Nurs..

[27] Joint Improvement Team Scotland Delayed Discharge. https://www.alliance-scotland.org.uk/blog/resources/joint-improvement-team-legacy-report/.

[28] Vesterby M.S., Pedersen P.U., Laursen M., Mikkelsen S., Larsen J., Søballe K., Jørgensen L.B. (2017). Telemedicine support shortens length of stay after fast-track hip replacement. Acta Orthop..

[29] Franck L.S., O’Brien K. (2019). The evolution of family-centered care: From supporting parent-delivered interventions to a model of family integrated care. Birth Defects Res..

[30] Clemente P.M., Pascual-Carrasco M., Hernandez C.M., de Molina R.M., Arvelo L.A., Cadavid B., Lopez F., Sanchez-Madariaga R., Sam A., Alonso A.T. (2021). Follow-up with Telemedicine in Early Discharge for COPD Exacerbations: Randomized Clinical Trial (TELEMEDCOPD-Trial). COPD.

[31] Kokorelias K.M., Gignac M.A.M., Naglie G., Cameron J.I. (2019). Towards a universal model of family centered care: A scoping review. BMC Heal. Serv. Res..

[32] Coffey A., Leahy-Warren P., Savage E., Hegarty J., Cornally N., Day M.R., Sahm L., O’connor K., O’doherty J., Liew A. (2019). Interventions to Promote Early Discharge and Avoid Inappropriate Hospital (Re)Admission: A Systematic Review. Int. J. Environ. Res. Public Health.

[33] Han E.-R., Yeo S., Kim M.-J., Lee Y.-H., Park K.-H., Roh H. (2019). Medical education trends for future physicians in the era of advanced technology and artificial intelligence: An integrative review. BMC Med Educ..

[34] Hoff T., Lee D.-R.D. (2022). Physician Satisfaction with Telehealth: A Systematic Review and Agenda for Future Research. Qual. Manag. Heal. Care.

[35] Bell-Aldeghi R., Gibrat B., Rapp T., Chauvin P., Le Guern M., Billaudeau N., Ould-Kaci K., Sevilla-Dedieu C. (2023). Determinants of the Cost-Effectiveness of Telemedicine: Systematic Screening and Quantitative Analysis of the Literature. Telemed. J. e-Health Off. J. Am. Telemed. Assoc..

