# Supplementary material for: Leveraging User-Friendly Mobile Medical Devices to Facilitate Early Hospital Discharges in a Pediatric Setting: A Randomized Trial Study Protocol
Source: Children (Basel). 2024 Jun 4;11(6):683. doi: 10.3390/children11060683 (PMC11201467; doi:10.3390/children11060683)
Supplement: Supplementary file 1 [file children-11-00683-s001.zip › children-2986521-supplementary.pdf]

DECLARATION OF CONFORMITY
European Medical Device Directive 93/42/EEC

Official Copy
18.09.2019

Manufacturer: **TytoCare Ltd.**

14 Beni Gaon Street

Netanya 4250803

Israel

Tel: + 972.72.2210750

Fax: + 972.72.2210752

We, Tyto Care Ltd., 14 Beni Gaon Street, Netanya 4250803, Israel, declare under our sole responsibility that the distributed CE marked product, conforms to the "CE Marking of Conformity Certificate", reference number D1412000005, firstly issued on June 15, 2018 under reference number D1412000003 and delivered by MDC - Medical Device Certification GmbH, Kriegerstraße 6, 70191 Stuttgart, Germany, Notified Body Identification Number 0483, fulfils the Annex I essential requirements of the Council Directive 93/42/EEC of 14 June 1993, concerning medical devices. Conformity is assured according to the guidelines set out in Annex II.

TytoCare Ltd. appointed MedNet as Authorized European Representative in the European Community located at:

MedNet GmbH

Borkstrasse 10,

48163 Muenster, Germany

Tel: +49 251 32266-61

Fax: +49 251 32266-22

This declaration of conformity is valid through the validity period of CE certificate.
The conformity to quality assurance set out in the said EN ISO 13485:2016 Conformity Certificate number D1412000006, issued and delivered by MDC - Medical Device Certification GmbH, Kriegerstraße 6, 70191 Stuttgart, Germany.

Product Name	Part number	Class	Put into service
**Tyto Stethoscope:**		class IIa, Annex IX (rule 10)	
Tyto Stethoscope tip	800-00001<br>800-00008		18 -09 -2019<br>18 -09 -2019
Tyto Device	800-00006<br>800-00013		18 -09 -2019<br>18 -09 -2019

Date: 18.09.2019
Place of issue: 14 Beni Gaon Street, Netanya, Israel

Signature:

Ofer Tzadik
COO
TytoCare Ltd.

DECLARATION OF CONFORMITY

European Medical Device Directive 93/42/EEC

Manufacturer:

TytoCare Ltd.

14 Beni Gaon Street

Netanya 4250803

Israel

Tel: + 972.72.2210750

Fax: + 972.72.2210752

Official Copy
18.09.2019

We, Tyto Care Ltd., 14 Beni Gaon Street, Netanya 4250803, Israel, declare under our sole responsibility that the distributed CE marked product, conforms to the "CE Marking of Conformity Certificate", reference number D1412000005, firstly issued on July 29, 2019 and delivered by MDC - Medical Device Certification GmbH, Kriegerstraße 6, 70191 Stuttgart, Germany, Notified Body Identification Number 0483, fulfils the Annex I essential requirements of the Council Directive 93/42/EEC of 14 June 1993, concerning medical devices. Conformity is assured according to the guidelines set out in Annex II.

TytoCare Ltd. appointed MedNet as Authorized European Representative in the European Community located at:

MedNet GmbH

Borkstrasse 10,

48163 Muenster, Germany

Tel: +49 251 32266-61

Fax: +49 251 32266-22

This declaration of conformity is valid through the validity period of CE certificate.
The conformity to quality assurance set out in the said EN ISO 13485:2016 Conformity Certificate number D1412000006, issued and delivered by MDC - Medical Device Certification GmbH, Kriegerstraße 6, 70191 Stuttgart, Germany.

Product Name	Part number	Class	Put into service
**Tyto Thermometer:**			
Tyto Device	800-00006<br>800-00013	class IIa, Annex IX (rule 10)	18 – 09 - 2019

Date: 18. 09.2019

Place of issue: 14 Beni Gaon Street, Netanya, Israel

Signature:

Ofer Tzadik
COO
TytoCare Ltd.

DECLARATION OF CONFORMITY

European Medical Device Directive 93/42/EEC

Manufacturer: **Tyto Care Ltd.**

14 Beni Gaon Street

Netanya 4250803

Israel

Tel: + 972.72.2210750

Fax: + 972.72.2210752

Official Copy
18.09.2019

We, Tyto Care Ltd., 14 Beni Gaon Street, Netanya 4250803, Israel, declare under our sole responsibility that the distributed CE marked product, meets the applicable harmonized standards and fulfils the Annex I essential requirements of the Council Directive 93/42/EEC of 14 June 1993, concerning medical devices. Conformity is assured according to the guidelines set out in Annex VII.

Tyto Care Ltd. appointed MedNet as Authorized European Representative in the European Community located at:

MedNet GmbH

Borkstrasse 10,

48163 Muenster, Germany

Tel: +49 251 32266-61

Fax: +49 251 32266-22

This declaration of conformity is valid through the validity of the declared models (part numbers) below.
The conformity to quality assurance set out in the said EN ISO 13485:2016 Conformity Certificate number D1412000006, issued and delivered by MDC - Medical Device Certification GmbH, Kriegerstraße 6, 70191 Stuttgart, Germany.

Product Name	Part number	Put into service
**Tyto Exam Camera** (class I, Annex IX (rule 12))		18 – 09 - 2019
Tyto Device	800-00013<br>800-00006	
Tongue depressor	800-00010<br>800-00004	
**Tyto Exam Camera Tongue Depressor Blades** (class I, Annex IX (rule 5))		
Child tongue depressor, 2 blades	435-00039	
Adult tongue depressor, 2 blades	435-00038	
Child tongue depressor blades, 10 (disposables)	435-00002	
Adult tongue depressor blades, 10 (disposables)	435-00003	
Child Tongue Depressor Blades, Gray, 100, Kit (disposable)	900-00009	
Adult Tongue Depressor Blades, Gray, 100, Kit (disposable)	900-00010	

Date: 18.09.2019

Place of issue: 14 Beni Gaon Street, Netanya, Israel

Signature:

Ofer Tzadik
COO
Tyto Care Ltd.

DECLARATION OF CONFORMITY

European Medical Device Directive 93/42/EEC

Official Copy 18.09.2019

Manufacturer: **Tyto Care Ltd.**

14 Beni Gaon Street

Netanya 4250803

Israel

Tel: + 972.72.2210750

Fax: + 972.72.2210752

We, Tyto Care Ltd., 14 Beni Gaon Street, Netanya 4250803, Israel, declare under our sole responsibility that the distributed CE marked product, meets the applicable harmonized standards and fulfils the Annex I essential requirements of the Council Directive 93/42/EEC of 14 June 1993, concerning medical devices. Conformity is assured according to the guidelines set out in Annex VII.

Tyto Care Ltd. appointed MedNet as Authorized European Representative in the European Community located at:

MedNet GmbH

Borkstrasse 10,

48163 Muenster, Germany

Tel: +49 251 32266-61

Fax: +49 251 32266-22

This declaration of conformity is valid through the validity of the declared models (part numbers) below.
The conformity to quality assurance set out in the said EN ISO 13485:2016 Conformity Certificate number D1412000006, issued and delivered by MDC - Medical Device Certification GmbH, Kriegerstraße 6, 70191 Stuttgart, Germany.

Product Name	Part number	Put into service
**Tyto Otoscope** (class I, Annex IX (rule 12))		18 – 09 - 2019
Tyto Device	800-00013<br>800-00006	
Tyto Otoscope tip	800-00002<br>800-00009	
**Disposable Ear Specula:** (class I, Annex IX (rule 5))		
Adult disposable ear specula, 5	435-00036	
Child disposable ear specula, 5	435-00037	
Adult disposable ear specula, 10	435-00005	
Child disposable ear specula, 10	435-00004	
Child Disposable Ear Specula, 100	900-00011	
Adult Disposable Ear Specula, 100	900-00012	

Date: 18.09.2019
Place of issue: 14 Beni Gaon Street, Netanya, Israel
Signature:

Ofer Tzadik
COO
Tyto Care Ltd.

CERTIFICATE

This is to certify that the Information Security Management System of

TYTO CARE LTD.

14, Beni Gaon St. , Netanya , Israel

Has been assessed and complies with the requirements of :

ISO/IEC 27001:2013

The Information Security Management System is Applicable to IT Operations Department Related to: Design, development, production and sales of devices and platform for the detection, measurement, recording and transfer of various physiological parameters to be used both by professionals and home users.

According to Statement of Applicability: Date: November 15, 2018.

**Initial Approval:**	25/12/2018		
**Issue Date:**	25/12/2018	**Revised:**	12/12/2019
**Valid Until:**	24/12/2021	**Certificate No.:**	96856

SII-QCD assumes no liability to any party other than the client, and then only in accordance with the agreed upon Certification Agreement. This certificate's validity is subject to the organization maintaining their system in accordance with SII-QCD requirements for system certification. The continued validity may be verified via scanning the code with a smartphone, or via website www.sii.org.il. This certificate remains the property of SII-QCD.

R.N 514545888

Avital Weinberg
Director, Quality & Certification Division

Our Vision: To Enhance Both Global Competitiveness of our Services, with our Uncompromised Quality and Integrity

THE INTERNATIONAL CERTIFICATION NETWORK

Digitaly Signed

CERTIFICATE

THE STANDARDS INSTITUTION OF ISRAEL

has issued an IQNet recognized certificate that the organization:

TYTO CARE LTD.

14, Beni Gaon St., Netanya, Israel

Has implemented and maintains a Information Security Management System that is Applicable For The Following scope of IT Operations Department Related to

Design, development, production and sales of devices and platform for the detection, measurement, recording and transfer of various physiological parameters to be used both by professionals and home users.

which fulfils the requirement of the following standard:

ISO/IEC 27001:2013

Issued on:	25/12/2018
Date of initial approval:	25/12/2018
Date of expiration:	24/12/2021

This attestation is directly linked to the IQNet Partner's original certificate and shall not be used as a stand-alone document

Registration number: IL - 96856

Alex Stoichitoiu
President of IQNet

Avital Weinberg
Director, Quality & Certification Division

IQNet Partners*:
AENOR *Spain* AFNOR Certification *France* APCER *Portugal* CCC *Cyprus* CISQ *Italy*
CQC *China* CQM *China* CQS *Czech Republic* Cro Cert *Croatia* DQS Holding GmbH *Germany* *EAGLE Certification Group USA*
FCAV *Brazil* FONDONORMA *Venezuela* ICONTEC *Colombia* Inspecta Sertifiointi Oy *Finland* INTECO *Costa Rica*
IRAM *Argentina* JQA *Japan* KFQ *Korea* MIRTEC *Greece* MSZT *Hungary* Nemko AS *Norway* NSAI *Ireland*
NYCE-SIGE México PCBC *Poland* Quality Austria *Austria* RR *Russia* SII *Israel* SIQ *Slovenia*
SIRIM QAS International *Malaysia* SQS *Switzerland* SRAC *Romania* TEST St Petersburg *Russia* TSE *Turkey* YUQS *Serbia*

* The list of IQNet partners is valid at the time of issue of this certificate. Updated information is available under www.iqnet-certification.com

www.sii.org.il

Certificate

mdc medical device certification GmbH
certifies that

Tyto Care Ltd.
14 Beni Gaon St.
Netanya, 4250803
Israel

for the scope

Design, development, production and distribution of devices for the detection, measurement, recording and transfer of various physiological parameters to be used both by professionals and home users

has introduced and applies a

Quality Management System

The mdc audit has proven that this quality management system meets all requirements of the following standard

EN ISO 13485

Medical devices – Quality management systems –
Requirements for regulatory purposes

EN ISO 13485:2016 + AC:2016 - ISO 13485:2016

Valid from	2019-07-29
Valid until	2022-07-28
Registration no.	D1412000006
Report no.	P19-00430-142812
Stuttgart	2019-07-29

Head of Certification Body

DAkkS
Deutsche Akkreditierungsstelle
D-ZM-16002-06-00

mdc medical device certification GmbH
Kriegerstraße 6
D-70191 Stuttgart, Germany
Phone: +49-(0)711-253597-0
Fax: +49-(0)711-253597-10
Internet: http://www.mdc-ce.de

For electronic publication only

EC Certificate

mdc medical device certification GmbH
Notified Body 0483
herewith certifies that

Tyto Care Ltd.
14 Beni Gaon St.
Netanya, 4250803
Israel

for the scope

Tyto Stethoscope
(electronic stethoscope enabling detection, measurement, recording and transfer of physiological parameters)
Tyto Thermometer

has introduced and applies a

Quality System

for the design, manufacture and final inspection.

The mdc audit has proven that this quality system meets all requirements according to

Annex II – excluding Section 4 of the Council Directive 93/42/EEC

of 14 June 1993 concerning medical devices.

The surveillance will be held as specified in Annex II, Section 5.

Valid from	2019-07-29
Valid until	2024-05-26
Registration no.	D1412000005
Report no.	P18-01395-131609
Stuttgart	2019-07-29

Head of Certification Body

medical device certification mdc

Benannt durch/Designated by
Zentralstelle der Länder für Gesundheitsschutz bei Arzneimitteln und Medizinprodukten
www.zlg.de
ZLG-BS-246.10.06

mdc medical device certification GmbH
Kriegerstraße 6
D-70191 Stuttgart, Germany
Phone: +49-(0)711-253597-0
Fax: +49-(0)711-253597-10
Internet: http://www.mdc-ce.de

For electronic publication only

ANNEX A

TYTO SOLUTION SPECIFICATIONS, PRICE LIST & WARRANTY PERIOD

[Reference from Sections 1.16, 7.1 & 14.1]

TYTO SOLUTION SPECIFICATIONS

**TytoCare**	
Dimensions	3.35 x 2.87 x 1.85 inch (85 x 73 x 47mm)
Weight	0.33 lbs. (0.15kg)
Display	2.4” LCD touch screen
Battery	Li-ion, built-in, rechargeable
Typical battery life	Up to 400 cycles of charge/discharge
Audio output port	Standard 3.5mm headphone connector
Power supply	Proprietary plug, 5Vdc@2A
**Tyto Camera**	
Max Sensor Resolution	5 MP
Max Video Resolution	640x480 (VGA)
Illumination	LED, Embedded
**Tyto Stethoscope**	
frequency range	20 - 3,500Hz
Heart rate range	30 - 250 BPM,
Audio output	3.5mm standard headphone connector
Dimensions	1.57 x 1.53 inch (40 x 39 mm)
Weight	0.13 lbs. (0.06kg)
**Tyto Otoscope**	
Image resolution	640x480 (VGA)
Tip diameter	0.16 inch (4.2mm)

ANNEX A

SPECIFICHE DI SOLUZIONE TYTO & PERIODO DI GARANZIA

[Riferimento dalle sezioni 1.16, 7.1 e 14.1]

SPECIFICHE DELLA SOLUZIONE TYTO

**TytoCare**	
Dimensioni	3,35 x 2,87 x 1,85 pollici (85 x 73 x 47 mm)
Peso	0,33 lbs. (0,15 kg)
Visualizzazione	2.4" LCD touch screen
Batteria	Ioni di litio, incorporato, ricaricabile
Durata tipica della batteria	Fino a 400 cicli di carica/scarico
Porta di uscita audio	Connettore per cuffie standard da 3,5 mm
Alimentazione	Spina proprietaria, 5Vdc@2°
**Fotocamera Tyto**	
Risoluzione massima del sensore	5 MP (in via in se del
Risoluzione video massima	640x480 (VGA)
Illuminazione	LED, Incorporato
**Stetoscopio di Tyto**	
intervallo di frequenza	20 - 3.500Hz
Intervallo frequenza cardiaca	30 - 250 BPM,
Uscita audio	Connettore per cuffie standard da 3,5 mm
Dimensioni	1,57 x 1,53 pollici (40 x 39 mm)
Peso	0,13 lbs. (0,06 kg)
**Otoscopio di Tyto**	
Risoluzione dell'immagine	640x480 (VGA)
Diametro della punta	0,16 pollici (4,2 mm)

Disposable speculum	Adult – 0.16inch (4mm); Child – 0.12 inch (3mm)
Weight	0.044 lbs. (0.02kg)
**Tyto Tongue Depressor**	
Reusable blade length	Adult (L8): 3.15inch (80mm); Child (L6): 2.36inch (60mm)
Weight	0.024 lbs. (0.011kg)
**Tyto Thermometer**	
Temperature range	34.4–42.2 °C (94.0–108.0 °F)
Laboratory Accuracy	0.2°C (0.4°F) accuracy for 38°C - 41°C (100.4°F - 105.8°F) range<br>0.3°C (0.5°F) accuracy outside the above range.<br>Complies with ASTM E1965-98 and ISO 80601-2-56
Resolution	0.01°F (0.01°C)
**Wireless Network**	
Wireless comm. standard	IEEE 802.11 N (recommended) \ G
Frequency band	2.4 GHz only *
Security protocols	WPA, WPA2 **
Encryption protocols	TKIP, AES

Speculum usa e getta	Adulto – 0.16inch (4mm); Bambino – 0,12 pollici (3mm)
Peso	0,044 lbs. (0,02 kg)
**Tyto Lingua Depressor**	
Lunghezza della lama riutilizzabile	Adulto (L8): 3.15inch (80mm); Bambino (L6): 2.36inch (60mm)
Peso	0,024 lbs. (0,011 kg)
**Termometro di Tyto**	
Intervallo di temperatura	(94,0–108,0 gradi centigradi)
Precisione di laboratorio	Precisione di 0,2 gradi centigradi per una gamma compresa tra 38 e 41 gradi centigradi<br>Precisione di 0,3 gradi centigradi al di fuori dell'intervallo precedente.<br>Conforme con ASTM E1965-98 e ISO 80601-2-56
Risoluzione	0.01oF (0. 01oC)
**Rete wireless**	
Standard di comunicazione wireless	IEEE 802.11 N (consigliato)
Banda di frequenza	Solo 2,4 GHz
Protocolli di sicurezza	WPA, WPA2
Protocolli di crittografia	TKIP, AES
Velocità di connessione	Consigliato:

Connection speed (relevant mainly for online)	Recommended: download: 15 Mbps \ Upload: 3 Mbps<br>Minimum: download: 5 Mbps \ Upload: 2Mbps
**Software**	
TytoCare App	Operating Systems:<br>• iOS 10 and up<br>• Android 4.4.4 and above<br>Apple models: iPhone 5S and up, iPad Air and up, iPad mini 3 and up, iPod touch 6 and up<br>Android models: Samsung Galaxy S5 and up, S6 plus, Note 5 and up, A7 and up, J7 and up, LG G4 and up
**Operating Conditions**	
Temperature	5 – 40 □C / 41 - 104 □F<br>For Tyto Thermometer: 16 – 40 □C (60.8 - 104 □F)
Humidity	15 - 70% (non-condensing)
**Storage Conditions**	
Temperature	-20 – 60 °C / -4 - 140□F
Humidity	15 - 70% (non-condensing)
Atmospheric pressure	700hPa to 1060hPa

(rilevante principalmente per online)	download: 15 Mbps e caricamento: 3 Mbps<br>Minimo: download: 5 Mbps e Upload: 2 Mbps
**Software**	
TytoCare App	Sistemi operativi:<br>• iOS 10 e fino<br>• Android 4.4.4 e versioni successive<br>Modelli Apple: iPhone 5S e oltre, iPad Air e fino, iPad mini 3 e oltre, iPod touch 6 e oltre<br>Modelli Android: Samsung Galaxy S5 e versioni successive, S6 plus, Nota 5 e oltre, A7 e versioni successive, J7 e versioni successive, LG G4 e versioni successive
**Condizioni di funzionamento**	
Temperatura	5 – 40oC / 41 - 104<br>Per il termometro Tyto: 16 – 40o C (60,8 - 104)
Umidità	15 - 70% (non condensante)
**Condizioni di conservazione**	
Temperatura	-20 – 60 gradi centigradi / -4 - 140
Umidità	15 - 70% (non condensante)
Pressione atmosferica	Da 700hPa a 1060hPa

TytoCare IT Architecture & Data Flow

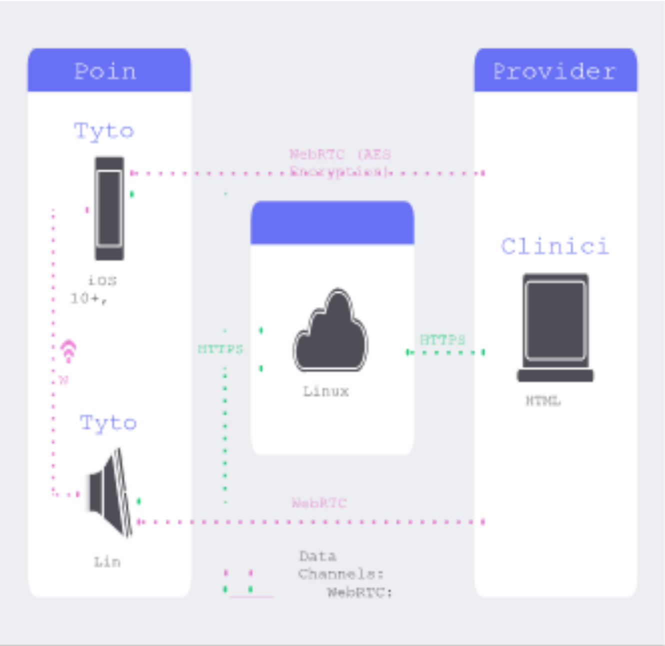

TytoCare App

Free App downloadable from the Apple and Google

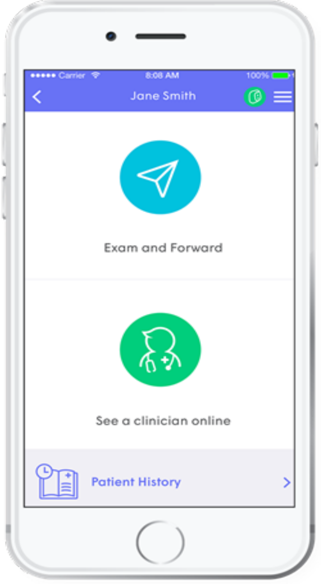

Play app stores. The TytoCare App authenticates the user prior to providing access to the App functionality.

The App enables the following capabilities:

- Initiating an online exam with a clinician including videoconference & exam data (synchronous).
- Send store-and-forward data to a clinician for review (asynchronous).
- Receive notifications and responses from clinicians (inbox).

The TytoCare App immediately uploads examination

Architettura IT di TytoCare e flusso di dati

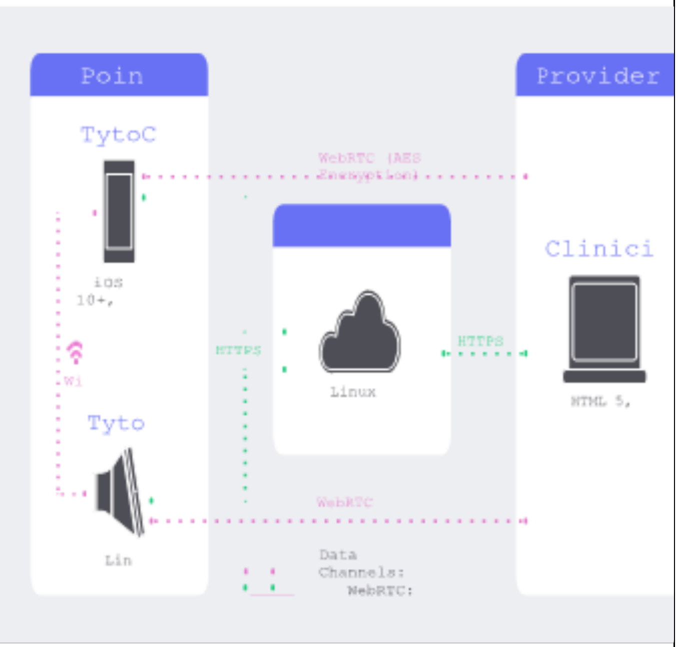

TytoCare App

App gratuita scaricabile dagli app store Apple e Google Play.

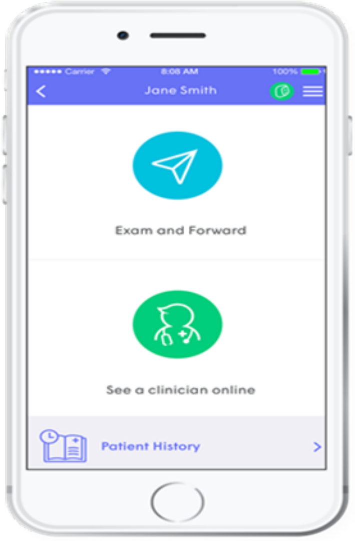

L'app TytoCare autentica l'utente prima di fornire l'accesso alla funzionalità dell'app.

L'app abilita le seguenti funzionalità:

- Avvio di un esame online con un medico che include videoconferenza e dati dell'esame (sincrono).
- Inviare i dati di archiviazione e inoltro a un medico per la revisione (asincrona).
- Ricevere notifiche e risposte dai medici (posta in arrivo).

L'app TytoCare carica immediatamente i dati

data received from the Tyto Device to the Tyto Cloud Server. After confirmation that the data resides in the Tyto Cloud Server, it is erased locally. In any case of disconnection from the Tyto Cloud Server, the examination data is immediately purged locally (from the App) until a reconnection is established with the Tyto Cloud Server.<br><br>**Tyto Device**<br>Used to perform the examinations, collect examination data and transfer it (via the Tyto Cloud Server) to the TytoCare App using TLS 1.2 encryption. The Tyto Device also streams the real time data to the TytoCare App and transmits short-time recordings to the Clinician App using a secured peer- to-peer protocol (WebRTC, utilizing DTLS).<br><br>Once the examination data has been securely transferred from the TytoCare App to the Tyto Cloud Server, the data is erased locally.<br>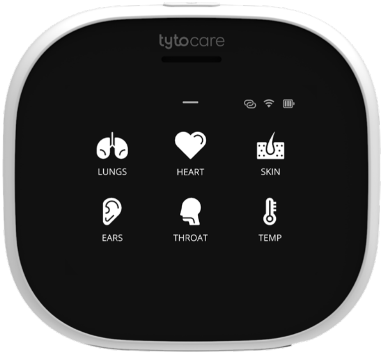<br><br><br>**Video Conference**	dell'esame ricevuti dal dispositivo Tyto al server Tyto Cloud. Dopo la conferma che i dati risiedono nel Tyto Cloud Server, vengono cancellati localmente. In ogni caso di disconnessione dal Tyto Cloud Server, i dati dell'esame vengono immediatamente eliminati localmente (dall'App) fino a quando non viene stabilita una riconnessione con il Tyto Cloud Server.<br><br>**Dispositivo Tyto**<br>Utilizzato per eseguire gli esami, raccogliere i dati degli esami e trasferirli (tramite il Tyto Cloud Server) all'app TytoCare utilizzando la crittografia TLS 1.2. Il dispositivo Tyto trasmette anche i dati in tempo reale all'app TytoCare e trasmette le registrazioni a breve tempo all'App Clinician utilizzando un protocollo peer-to-peer protetto (WebRTC, utilizzando DTLS).<br><br>Una volta che i dati dell'esame sono stati trasferiti in modo sicuro dall'app TytoCare al Tyto Cloud Server, i dati vengono cancellati localmente.<br>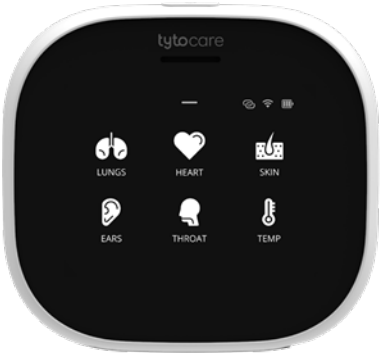<br><br><br>**Videoconferenza**

The videoconference is conducted over WebRTC (peer-to-peer protocol). WebRTC is a secured protocol which utilizes SRTP, SCTP and DTLS for the streams, control and data channels.<br><br>If the Firewall (NAT configuration) permits UDP pass-through then a peer-to-peer connection is enabled (lowest latency).<br><br>If a symmetrical NAT is configured in the Firewall, a relayed connection is required via the Tyto Server. When a relayed connection is utilized, the TytoCare platform provides dedicated servers for media stream relay.	La videoconferenza è condotta su WebRTC (protocollo peer-to-peer). WebRTC è un protocollo protetto che utilizza SRTP, SCTP e DTLS per i flussi, il controllo e i canali di dati.<br><br>Se il firewall (configurazione NAT) consente UDP pass-through quindi una connessione peer-to-peer è abilitata (latenza più bassa).<br><br>Se nel firewall è configurato un NAT simmetrico, è necessaria una connessione inoltrata tramite il server Tyto. Quando viene utilizzata una connessione inoltrata, la piattaforma TytoCare fornisce server dedicati per l'inoltro del flusso multimediale.
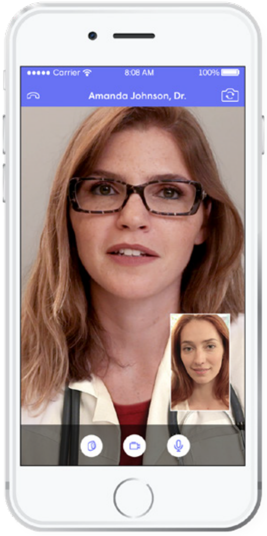	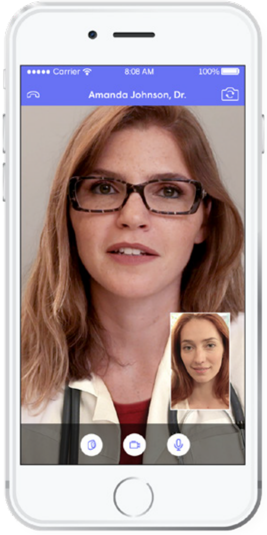
**Clinician App**<br>The Clinician App is a secure browser based HTML 5 web app. In order to fully utilize all the capabilities of the Clinician App, it is required to use a device running on a Microsoft Windows or macOS operating system with a Google Chrome browser, as it supports WebRTC (videoconferencing component) and in-browser audio playback natively. The Clinician App authenticates the user prior to providing access to the App functionality.<br><br>The Clinician App enables the following capabilities:<br>• Conduct online exams (synchronous).<br>• Respond to patient store-and-forward requests (asynchronous).<br>• Review exam history (inbox).	**App Clinica**<br>L'app clinica è un'app web HTML 5 basata su browser sicuro. Al fine di utilizzare appieno tutte le funzionalità dell'App Clinica, è necessario utilizzare un dispositivo in esecuzione su un sistema operativo Microsoft Windows o macOS con un browser Google Chrome, in quanto supporta WebRTC (componente di videoconferenza) e la riproduzione audio in-browser in modo nativo. L'App Clinica autentica l'utente prima di fornire l'accesso alla funzionalità dell'app.<br>L'App Clinica consente le seguenti funzionalità:<br>• Condurre esami online (sincroni).<br>• Rispondere alle richieste di archiviazione e inoltro dei pazienti (asincrone).<br>• Rivedere la cronologia degli esami (posta in arrivo).<br>•<br>L'App Clinica non memorizza alcun dato localmente (browser). L'app clinica viene distribuita su una rete

The Clinician App does not store any data locally (browser). The Clinician App is deployed on a

CDN (AWS Cloud Front) to enable fast and secure access.

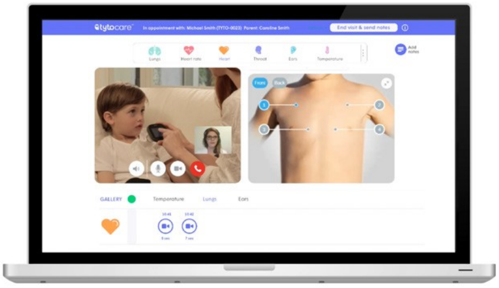

Tyto Cloud Server

The Tyto Cloud Server is fault-tolerant, stress-tested and fully redundant, running on Amazon Web Services (AWS) with the following topology:

- Dedicated VPC divided into three subnets
- Completely managed by ACL and security groups and based on machine roles (access keys do not leave the VPC)
- Utilizing AWS elastic beanstalk – Linux based machines running Tomcat that are monitored, updated and auto-scale automatically

TytoCare AWS account is secure via a two-factor authentication process:

- Server access requires an access token which is generated to the end-user only after authentication using username / password.
- The Tyto Cloud Server manages patient information only via HIPAA compliant services such as RDS, S3, EC2 (running elastic beanstalk) and the elastic load balancer. The Tyto Cloud Server database guarantees data / transaction completeness. AWS RDBMS (RDS) provides built-in mechanisms for availability, scalability, durability and security.

CDN (AWS Cloud Front) per consentire un accesso rapido e sicuro.

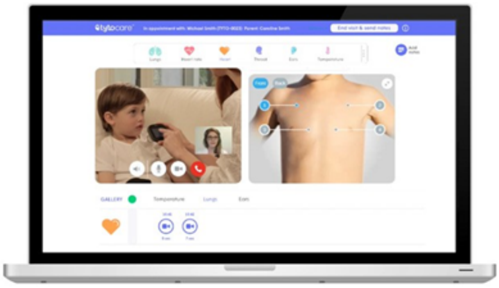

Tyto Cloud Server

Tyto Cloud Server è a tolleranza d'errore, sottoposto a stress e completamente ridondante, e viene eseguito su Amazon Web Services (AWS) con la topologia seguente:

- VPC dedicato diviso in tre subnet
- Completamente gestito da ACL e gruppi di sicurezza e in base ai ruoli computer (i tasti di accesso non lasciano il VPC)
- Utilizzo di AWS elastic beanstalk: macchine basate su Linux che eseguono Tomcat che vengono monitorate, aggiornate e scalabili automaticamente

L'account AWS di TytoCare è sicuro tramite un processo di autenticazione a due fattori:

- L'accesso al server richiede un token di accesso che viene generato all'utente finale solo dopo l'autenticazione utilizzando nome utente / password.
- Tyto Cloud Server gestisce le informazioni sui pazienti solo tramite servizi conformi a HIPAA come RDS, S3, EC2 (running elastic beanstalk) e il bilanciamento del carico elastico. Il database di Tyto Cloud Server garantisce la completezza dei dati/transazioni. AWS RDBMS (RDS) fornisce meccanismi integrati per disponibilità, scalabilità, durata e sicurezza.

Sicurezza e privacy:

**Security & Privacy:**<br><br>**Data Encryption**<br>TytoCare is using Advanced Encryption Standard (AES) 256-bit encryption on both the recordings & metadata stored in AWS (Amazon Web Services). In 2003, the US government announced that AES could be used to protect classified information.<br><br>**Data Repository Location & Security**<br>The data collected by TytoCare is stored on AWS dedicated & HIPAA/GDPR compliant services for storage (S3) and database (RDS). These AWS services guarantee that data-at-rest is kept encrypted. TytoCare’s production environment resides on Amazon US East region (North Virginia), Canada and EU (Ireland).<br><br>**Product HIPAA & GDPR Compliance**<br>TytoCare is utilizing HIPAA & GDPR compliant AWS services. Additionally, TytoCare has implemented a technical safeguards review (HIPAA, GDPR) on the platform and has aligned its product features accordingly:<br>• Audit Trail monitoring any access to PHI<br>• HIPAA & GDPR compliant user authentication mechanism, including password policies & session restrictions (e.g. auto log-off)<br>• PHI security and integrity mechanisms	**Crittografia dei dati**<br>TytoCare utilizza la crittografia a 256 bit di Advanced Encryption Standard (AES) sia per le registrazioni che per i metadati archiviati in AWS (Amazon Web Services). Nel 2003, il governo degli Stati Uniti ha annunciato che AES potrebbe essere utilizzato per proteggere le informazioni classificate.<br><br>**Posizione e sicurezza dell'archivio dati**<br>I dati raccolti da TytoCare vengono archiviati su servizi conformi HIPAA/GDPR per storage (S3) e database (RDS) di AWS dedicati e HIPAA/GDPR. Questi servizi AWS garantiscono che i dati inattivi vengano mantenuti crittografati. L'ambiente di produzione di TytoCare risiede nella regione di Amazon US East (Virginia settentrionale), Canada e UE (Irlanda).<br><br>**Conformità HIPAA e GDPR del prodotto**<br>TytoCare utilizza i servizi AWS conformi a HIPAA e GDPR. Inoltre, TytoCare ha implementato una revisione delle misure tecniche di salvaguardia (HIPAA, GDPR) sulla piattaforma e ha allineato le sue caratteristiche di prodotto di conseguenza:<br>• Audit Trail monitora qualsiasi accesso a PHI<br>• Meccanismo di autenticazione utente conforme a HIPAA e GDPR, inclusi i criteri password e le restrizioni di sessione (ad esempio la disconnessione automatica)<br>• Meccanismi di sicurezza e integrità PHI
